# Ultrastructural Features of Gold Nanoparticles Interaction with HepG2 and HEK293 Cells in Monolayer and Spheroids

**DOI:** 10.3390/nano10102040

**Published:** 2020-10-16

**Authors:** Boris Chelobanov, Julia Poletaeva, Anna Epanchintseva, Anastasiya Tupitsyna, Inna Pyshnaya, Elena Ryabchikova

**Affiliations:** Institute of Chemical Biology and Fundamental Medicine, Siberian Branch of Russian Academy of Science, Lavrent’ev av., 8, 630090 Novosibirsk, Russia; boris.p.chelobanov@gmail.com (B.C.); fabaceae@yandex.ru (J.P.); annaepanch@gmail.com (A.E.); aysa@ngs.ru (A.T.); pyshnaya@niboch.nsc.ru (I.P.)

**Keywords:** AuNPs, AuPEI-NPs, AuBSA-NPs, electron microscopy, ultrastructure of HepG2 cells and spheroids, ultrastructure of HEK293 cells and spheroids, penetration of NPs into monolayer and spheroids

## Abstract

Use of multicellular spheroids in studies of nanoparticles (NPs) has increased in the last decade, however details of NPs interaction with spheroids are poorly known. We synthesized AuNPs (12.0 ± 0.1 nm in diameter, transmission electron microscopy (TEM data) and covered them with bovine serum albumin (BSA) and polyethyleneimine (PEI). Values of hydrodynamic diameter were 17.4 ± 0.4; 35.9 ± 0.5 and ±125.9 ± 2.8 nm for AuNPs, AuBSA-NPs and AuPEI-NPs, and Z-potential (net charge) values were −33.6 ± 2.0; −35.7 ± 1.8 and 39.9 ± 1.3 mV, respectively. Spheroids of human hepatocarcinoma (HepG2) and human embryo kidney (HEK293) cells (Corning ® spheroid microplates CLS4515-5EA), and monolayers of these cell lines were incubated with all NPs for 15 min–4 h, and fixed in 4% paraformaldehyde solution. Samples were examined using transmission and scanning electron microscopy. HepG2 and HEK2893 spheroids showed tissue-specific features and contacted with culture medium by basal plasma membrane of the cells. HepG2 cells both in monolayer and spheroids did not uptake of the AuNPs, while AuBSA-NPs and AuPEI-NPs readily penetrated these cells. All studied NPs penetrated HEK293 cells in both monolayer and spheroids. Thus, two different cell cultures maintained a type of the interaction with NPs in monolayer and spheroid forms, which not depended on NPs Z-potential and size.

## 1. Introduction

Gold nanoparticles have a number of unique physical and chemical properties that, together with a good biocompatibility, makes them a promising tool for nanomedicine. Advantages of using gold nanoparticles (AuNPs) and their various modifications in the treatment and diagnosis of diseases are being actively studied; a number of comprehensive detailed reviews is devoted to this issue [1,2,3,4,5,6]. Similar to other NPs, AuNPs are studied in cell cultures and in laboratory animals; and in last decade a new experimental model has been developed: multicellular spheroids or micro-tissues (cell cultures in 3D-form); the advantages of spheroids are described in details [7,8,9,10]. Spheroids that mimic the structure and functions of various tissues have shown their suitability for studies of different problems in modern biomedicine, including the effects of drugs, drug damage to the liver, toxicity of chemical compounds, and human hepatocarcinoma (HepG2) spheroids are considered in such studies as a practically adequate replacement of primary hepatocytes [11,12,13,14,15]. The advent of commercially available devices for cultivation of spheroids has transformed their obtaining from "high art" into affordable technology, which expanded the scope of their application. Various approaches for obtaining spheroids are reported, which roughly can be divided to scaffold-based and scaffold-free; see reviews [8,12,16,17,18].

The number of published works on cellular spheroids is already in the thousands, but many details of their structure remain unknown, including the structure of their external surface and the morphological substrate of contact with the environment. Meanwhile, structure of the region adjacent to spheroids surface determine the nature of interaction not only with the culture medium, but also with soluble preparations and NPs containing in that medium. Morphological changes in spheroids treated with NPs or chemical compounds are studied mainly in transmitted light and various fluorescence methods [9,19,20,21]. The use of electron microscopy is rare and mostly is limited to registration of NPs presence in a cell [22,23,24] or TEM-illustration of NPs used in a study [9,15,21,25,26]. However, the size of NPs requires studying their interaction with cells at subcellular level, which is realized in a transmission electron microscopy (TEM) of ultrathin sections.

In this work, we examined and compared the morphology of HepG2 and human embryo kidney (HEK293) cell monolayers and spheroids with TEM and scanning electron microscopy (SEM), because we found out an insufficiency of published data. Both cell lines are epithelial in nature; however, HepG2 is well-differentiated line, which possesses structural and morphologic characteristics of hepatocytes, while morphology of HEK 293 cell line does not show tissue-specific features. In this work, we describe structural organization of the spheroids and point out the features specific for each cell type.

It was interesting to find out how HepG2 and HEK293 epithelial cell lines interact with the same NPs in monolayer and spheroids. We incubated the cells in monolayer and spheroids with synthesized AuNPs and their modified variants coated with protein (bovine serum albumin, BSA) or polymer (polyethylenimine, PEI). Here, we present data on features of the penetration of these NPs into HepG2 and HEK293 cells and compare those in spheroids and monolayer.

In common, in this work we present new comparative data on morphology of HepG2 and HEK293 cells in monolayer and spheroids, and their interaction with AuNPs, AuPEI and AuBSA-NPs.

## 2. Materials and Methods

### 2.1. Reagents

All used reagents were analytical grade. Ultrapure water of 18.2 MΩ·cm at 25 °C (Simplicity 185 system, Millipore, Burlington, MA, USA) was used in all processes of NPs preparation.

### 2.2. Preparation of AuNPs, AuBSA-NPs and AuPEI-NPs

AuNPs were synthesized similarly to [27]. In brief, the solution of Na_3_C_6_H_5_O_7_⋅3H_2_O (5 mL, 38.8 mM) (Fluka, Charlotte, NC, USA) was added under stirring to the boiled solution of HAuCl_4_⋅3H_2_O (45 mL, 1 mM) (Aurat, Moscow, Russia). The mixture was intensively stirred for 20 min and kept at room temperature for 24 h, and then filtered (pore size of 0.45 μm, MDI, Ambala Cantt, India). Extinction coefficient of resultant suspension was ε_260_ = 8.78 × 10^8^ M^–1^ cm^–1^ (Shimadzu, Kyoto, Japan), which corresponds to a concentration of 3.6 × 10^–9^ M of Au [28]. The suspension was stored at 4 °C.

AuPEI-NPs were prepared by layer-by-layer approach. Initially, the reaction mixture (695 μL) containing AuNPs (3.6 nM) and 0.72 μM oligodeoxyribonucleotide (ON) was incubated for 30 min at 56 °C to prepare non-covalent AuON-NPs serving as an intermediate compound [29]. Oligodeoxyribonucleotide (5′-TTT TTT TTT TTT TTT TTT TTT TTT TT-3′) was synthesized on an ASM-800 (Biosset, Novosibirsk, Russia) by the solid-phase phosphoroamidite protocol using phosphoramidites from ChemGenes (Wilmington, MA USA). The ON was purified by reversed phase high performance liquid chromatography (HPLC) on an Agilent 1200 Series (Santa Clara, CA, USA) using a Zorbax 5 μm Eclipse-XDB-C18 80 Å column (150 × 4.6 mm^2^) by Agilent (Santa Clara, CA, USA).

The AuON-NPs were washed with 0.5 mL of 4 mM Na_3_C_6_H_5_O_7_ solution and precipitated by centrifugation for 30 min at 13,200 rpm. The precipitate was diluted with 0.57 mL of 4 mM Na_3_C_6_H_5_O_7_ solution, and pH of the suspension was adjusted to 10 with 12.5 μL of 1 M Na_2_HPO_4_ (AlfaChem Plus, Saint Petersburg, Russia). Solution of 100 μL of 0.8% 11-mercaptoundecanoic acid (MUA) (Sigma-Aldrich, St. Louis, MO, USA) in 10% ethanol (Kemerovo Pharmaceutical factory, Kemerovo, Russia) was added with shaking (1400 rpm) to AuON-NPs, and the mixture was incubated for 30 min at 25 °C to obtain AuON-MUA-NPs. The product was washed with 0.6 mL of 1 mM NaCl (Panreac, Barcelona, Spain), and precipitated by centrifugation for 10 min at 13,200 rpm. The precipitate was diluted with 0.25 mL of 1 mM NaCl. The final step was carried out in several tubes (Eppendorf, Hamburg, Germany). The AuON-MUA-NPs (50 μL) were added with shaking (1400 rpm) to 50 μL of 0.1% branched polyethylenimine (PEI) in each tube. The PEI (-NHCH_2_CH_2_-)_x_(-N(CH_2_CH_2_NH_2_)CH_2_CH_2_-)y, 10 kDa of molecular weight, was 99% of purity (Alfa Aesar, Ward Hill, MA, USA). The mixture was incubated for 30 min at 25 °C. Resulting product was washed with 0.5 mL of 1 mM NaCl, and then precipitated by centrifugation for 10 min at 13,200 rpm. The precipitate was diluted in 50 μL of 1 mM NaCl. The concentration of AuON-MUA-PEI-NPs (further designated as AuPEI-NPs) in the resulting suspension was 10 nM of AuNPs.

AuNPs coated with BSA were obtained by incubation of 250 μL 3 nM AuNPs with 50 μL of 10% BSA (Sigma, St. Louis, MO, USA) for 24 h on a Multi-rotator Multi Bio RS-24 at 10 rpm (Biosan, Riga, Latvia) [30]. The resulting AuBSA-NPs were washed with 1 mL of PBS (Sigma-Aldrich, St. Louis, MO, USA) and separated from the excess BSA by centrifugation for 30 min at 13,000 rpm on a Heraeus Biofuge pico (Thermo Fisher Scientific, Waltham, MA, USA). The AuBSA-NPs precipitate was suspended in PBS and the volume was brought to a concentration of AuNPs 10 nM. The stability of the AuBSA-NPs was confirmed by the absence of color changes when adding an equal volume of 3 M NaCl. The preparation was stored at 4 °C.

### 2.3. Physicochemical Characterization of Nanoparticles

All prepared NPs were examined in transmission electron microscope (TEM) (see Section 2.6). Optical extinction spectra were recorded on a Clariostar plate fluorimeter (BMG, Labtech Ortenberg, Germany) in the range of 400–800 nm according to manufacturer’s instructions.

Hydrodynamic characteristics of the NPs were evaluated by method of photon correlation spectroscopy on a Malvern Zetasizer Nano-ZS instrument (Malvern Instruments, Malvern, UK). The measurements were performed at least 5 times for each sample.

All prepared NPs were subjected to agarose gel electrophoresis. Samples containing 5 μL (0.5 pmol) of each kind of NPs and 1 μL glycerol/water (1:1, v/v) were loaded into the wells of 0. 8% agarose (Lonza Rockland, ME, USA) in Tris-Glycine buffer (250 mM glycine, 25 mM Tris, pH 8.3). The electrophoresis was carried out for 30 min at 5 V cm^−1^. Images were scanned using Epson Perfection 4990 Photo scanner (Seiko Epson Corporation, Suwa, Japan).

### 2.4. Cell Cultures and Spheroid Production

Cell cultures of human hepatocarcinoma (HepG2) and human embryo kidney (HEK293) cells were obtained from the Russian collection of cell cultures (Institute of Cytology RAS, Saint Petersburg, Russia). The monolayers were cultured in IMDM (HepG2) or DMEM (HEK 293) media, containing 10% embryonic calf serum (Thermo Fisher Scientific, Waltham, MA, USA) and 100 u/mL of penicillin and streptomycin (Thermo Fisher Scientific, Waltham, MA, USA) in an atmosphere of 5% CO_2_ at 37 °C.

To obtain spheroids (3D culture), Corning ^®^ spheroid microplates 96 well black/clear bottom round ULA (Ultra-Low Attachment surface) (CLS4515-5EA) (Corning, Corning, NY, USA) were used. Cells of the HepG2 and HEK 293 lines were seeded at a dose of 300 and 600 cells, correspondingly, per well. The spheroids were cultured for 7 days with daily imaging using a ZEISS Axiovert 200 m microscope (Carl Zeiss AG, Oberkochen, Germany), equipped with an AxioCam MRm camera (Carl Zeiss AG, Oberkochen, Germany) and a CO_2_ Incubator XL-3 (PeCon GmbH, Erbach, Germany). Measurements of spheroids were performed using AxioVision program.

### 2.5. Incubation of Cells and Spheroids with the NPs

Cells of the HepG2 and HEK293 lines were sown in Petri dishes (40 mm diameter, TPP Techno Plastic Products AG, Trasadingen, Switzerland), 10^5^ cells per dish. After reaching 70% coverage, the monolayers were washed with a culture medium, and AuNPs or AuBSA-NPs, or AuPEI-NPs suspended in an IMDM (for HepG2) or DMEM (for HEK293) were added to cells. Final concentration of AuNPs in the medium was 1 nM.

The cells were incubated for 15 min, 30 min, 1, 2 and 4 h in a medium without serum. Then the cells were rinsed three times with PBS, removed with trypsin, sedimented by centrifugation (5 min at 3000 rpm), and fixed with 4% paraformaldehyde for TEM studies.

Seven-day spheroids of HepG2 and HEK 293 cells cultured in 96-well plates were washed with a culture medium. The AuNPs or AuBSA-NPs or AuPEI-NPs were added to spheroids in an IMDM (HepG2-spheroids) or DMEM (HEK293 spheroids); final concentration of AuNPs was 1 nM. The spheroids were incubated with NPs for 1, 2 and 4 h without serum, and then fixed with 4% paraformaldehyde.

### 2.6. TEM Studies of NPs

Suspension of AuNPs was adsorbed on formvar-coated copper grids for 1 min, then liquid excess was removed by a filter paper, and the grid was air dried. Suspensions of AuNPs-PEI and AuNPs-BSA also were adsorbed on a grid for 1 min, and after removing of liquid excess were contrasted with 2% phosphotungstic acid (EMS, Hatfield, PA, USA), pH 0.5. The samples were examined with a JEM 1400 TEM (JEOL, Japan) equipped with a Veleta digital camera (EM SIS, Muenster, Germany). iTEM program, version 5.2 (EM SIS, Muenster, Germany) was used for direct measurement of NPs sizes.

### 2.7. TEM studies of Cell Cultures and Spheroids

All reagents for microscopic studies were purchased from EMS (Hatfield, PA, USA).

Samples of fixed cell cultures and spheroids were washed from paraformaldehyde with Hank’s balanced solution and were postfixed with 1% osmium tetraoxide solution for 1 h, dehydrated in ethanol and acetone according to the standard method, and then embedded in an epon-araldite mixture to obtain hard blocks.

Ultrathin and semithin sections were prepared on an ultramicrotome EM UC7 (Leica, Wetzlar, Germany) using a diamond knife (Diatome, Nidau, Switzerland). The semithin sections of spheroids were stained with Azur II and were examined in a Leica DM 2500 light microscope (Leica, Wetzlar, Germany) to choose an area for ultrathin sectioning. Ultrathin sections were contrasted with 2% water solutions of uranyl acetate and lead citrate and examined in a JEM 1400 TEM (JEOL, Japan). Digital images were collected using a Veleta side-mounted camera (EM SIS, Muenster, Germany).

### 2.8. Scanning Electron Microscopy

The seven-day spheroids of HepG2 and HEK 293 cells were fixed with a 4% paraformaldehyde at 4 °C for 24 h. Fixed spheroids were rinsed with PBS (Sigma-Aldrich, St. Louis, MO, USA), dehydrated using a graded ethanol series (50%, 70%, 80%, 90%, 96% and 100%) and then immersed to mixture of ethanol and hexamethyldisilazane (HMDS; Sigma-Aldrich, St. Louis, MO, USA) in a ratio 1:1 for 10 min, and then to 100% HMDS for 10 min. Spheroids were fixed on a sample stand using double-sided carbon tape and dried overnight in air. Spheroids were sputter coated with 10 nm gold/palladium and analyzed using a scanning electron microscope EVO 10 (Carl Zeiss AG, Oberkochen, Germany) at an accelerating voltage of 10 kV.

## 3. Results

### 3.1. Physicochemical Characteristics of the NPs

**Figure 1:**
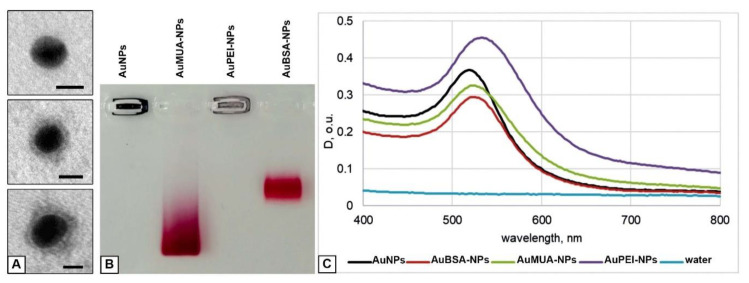
Physicochemical characterization of the NPs. (**A**) TEM images of AuNP (top), AuPEI-NP (middle) and AuBSA-NP (bottom). Negative staining with phosphotungstic acid. Bars correspond to 10 nm. (**B**) Image of the gel after electrophoretic analysis of AuNPs, AuMUA-NPs, AuPEI-NPs and AuBSA-NPs. (**C**) Optical extinction spectra of AuNPs, AuMUA-NPs, AuPEI-NPs and AuBSA-NPs in water. We used samples with different concentrations to separate the curve maximums for clear visualization.

We prepared AuNPs and covered them with PEI or BSA for cell studies. Values of polydispersity indexes evidenced that NP preparations represent well dispersed aqueous suspensions (Table 1). Examination in TEM revealed spherical naked particles of high electron density (d = 12.0 ± 0.1 nm) in sample of AuNPs, and the same particles surrounded with “corona” of PEI or BSA having middle electron density, in the samples of AuPEI-NPs and AuBSA-NPs (Figure 1A).

To prepare AuPEI-NPs we modified previously described layer-by-layer method, which uses MUA for stabilization of AuNPs [31]. We introduced a step of AuNPs preliminary incubation with ON (anyone with length 20–30 n.) [29]. This ON layer allowed to increase colloid stability of the NPs at subsequent stages of interaction with MUA and PEI. The resulting AuPEI-NPs had a positive net charge, unlike AuNPs and AuBSA-NPs, which have a negative net charge (Zeta potential) (Table 1). Optical extinction spectra did not critically change after covering of AuNPs with PEI or BSA (Figure 1C).

Thus, we obtained three types of well dispersed NPs to study their interaction with cells: AuNPs, AuPEI-NPs and AuBSA-NPs, similar by size, and differing in the magnitude of net charge (Table 1, Figure 1B). Detailed presentation of physico-chemical properties of NPs, similar to studied in this work could be found in our previous studies [32,33]. Absence of noticeable cytotoxicity of all studied NPs was shown in our previous work using two different cell cultures [32].

### 3.2. Cell Experimental Models

#### 3.2.1. Monolayer Cell Cultures

The well-differentiated hepatoma cell line HepG2, which has a wide set of properties inherent to human hepatocytes in vivo, has been actively used for about 40 years in various studies exploiting organ-specific features of the line [10,11,34,35,36,37]. Morphology of HepG2 cells was shown to keep main features of the hepatocytes: tight junctions separate apical and basolateral plasmalemma providing formation of bile capillaries and blood-biliary barrier; ultrastructural observations were supported by immunohistochemical and biochemical studies [38,39]. Based on current knowledge about the role of tight junctions in hepatic physiology and pathology, it is possible to claim that formation of these structures by HepG2 cells evidences for high levels of differentiation [40].

**Figure 2:**
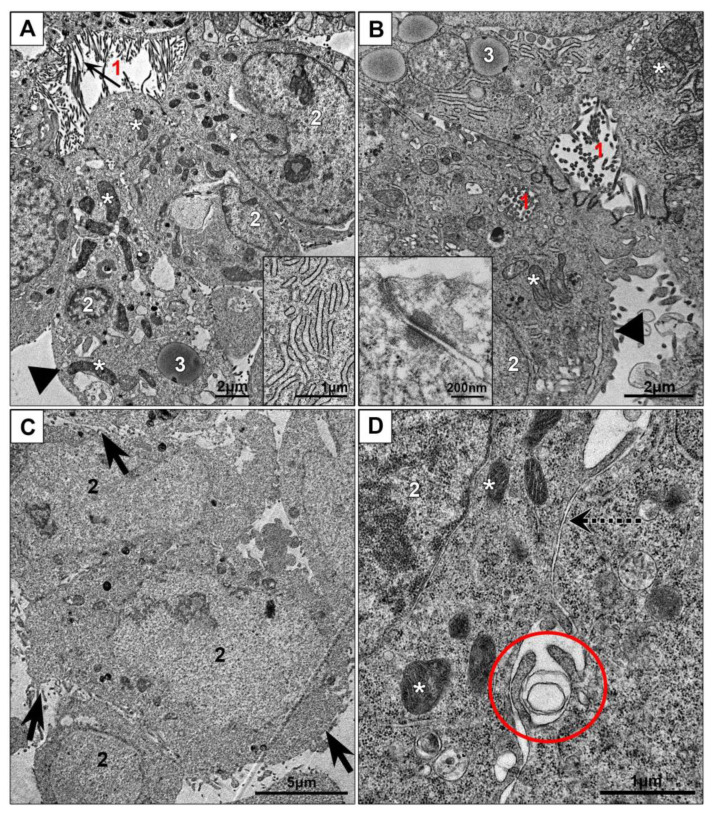
Cell cultures in monolayer: (**A**,**B**)—HepG2, (**C**,**D**)—HEK293. Inserts: (**A**) cisternae of endoplasmic reticulum; (**B**) tight junction and desmosome between cells at apical pole. 1—“bile capillaries”, arrows show microvilli; 2—nucleus; 3—lipid droplet; asterisks show mitochondria; arrowheads show basolateral membranes; dotted arrow shows area of “simple” contact of two cells; oval shows site of macropinocytosis; tick arrows show cell surface with many outgrowths.

Our examination of HepG2 cells on ultrathin sections revealed formation of bile capillaries that carry microvilli on the surface and had tight junctions between the cells; desmosomes connecting cell lateral surfaces were occasionally observed (Figure 2A,B). The cells were filled with granular cytoplasm containing mitochondria and cisternae of endoplasmic reticulum covered with ribosomes. Relatively small Golgi apparatus usually was located in perinuclear region. Many cells contained lipid droplets of medium electron density. Basolateral cell surfaces were mostly flat, some small outgrowths of cytoplasm of various shapes were observed (Figure 2A,B). Our study clearly showed that monolayer HepG2 cells retain characteristics of hepatocyte unique polarity, which differ from those in other types of epithelia [39,41].

HEK 293 cell line also was used in various studies for about 40 years for examining molecular characteristics of different cellular processes, endocytosis, gene expression, for transfection and production of proteins and lentiviral vectors for pharmaceutical industry and science [42,43,44]. HEK293 cells were obtained from human embryo kidney [42] and have “columnar” polarity, which is typical for non-hepatic epithelia [39,41].

HEK293 cells in monolayer were arranged in a disordered manner, tight junctions were not observed (Figure 2C,D). Cell surface was covered with numerous outgrowths; some of them were long and evidenced for macropinocytosis. On the sections, extended areas of cell close contact were observed, the distance between cell surfaces was about 10 nm, however, no interdigitations and tight junctions were present, which indicates absence of cell integration into “epithelial” layer. The cytoplasm contained numerous polysomes; mitochondria and endoplasmic reticulum cisternae were scarce (Figure 2C,D). In contrast to HepG2 cells, HEK293 monolayer cells did not possess signs of organ specialization.

#### 3.2.2. Spheroids of HepG2 and HEK293 Cells

Previously published studies showed changes in the shape and compactization of HepG2 spheroids during their development; early, middle and late stages of spheroid growth were identified, the duration of which varied in different works and mainly depended on the dose of cell seeding. Common recommendation was to work with HepG2 spheroids at middle stage (beginning from days 4–5 after cell seeding) which is characterized by active function of spheroid cells and absence of cell destruction [10,14,45]. Amount of published studies using HEK293 spheroids is incomparably less than studies with HepG2 spheroids; however, authors noted a very fast compactization of HEK293 cells on a non-adhesive surface and regular spherical shape of the spheroids [46,47].

**Figure 3:**
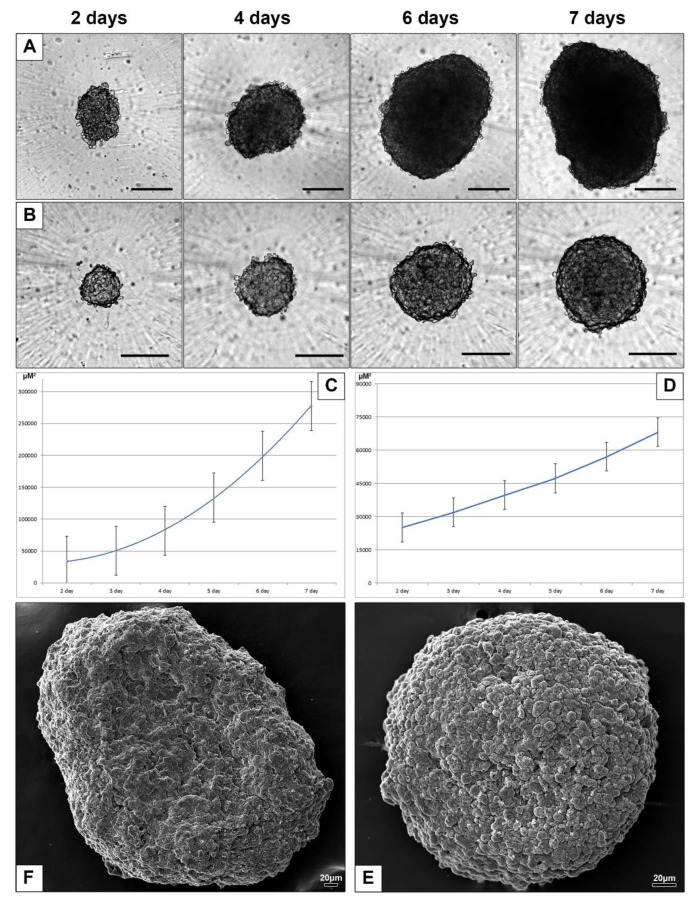
Characteristics of spheroids in culture. Representative light microscopic images of HepG2 spheroids (**A**) and HEK293 spheroids (**B**), days 2–7 after seeding. Bars correspond to 200 µm. Growth curves of spheroids square: (**C**) HepG2, (**D**) HEK293. Representative images of 7-day HepG2 (**E**) and HEK293 (**F)** spheroids obtained with SEM.

To examine interaction of AuNPs, AuPEI-NPs and AuBSA-NPs with HepG2 and HEK293 cells in 3D-culture we used corresponding spheroids cultured for 7 days with daily monitoring and photographing. In the first three days after cell seeding HepG2 spheroids looked loose and had an irregular shape, while HEK293 spheroids became spherical within a day. The spheroids showed differences in shape and rate of growth (Figure 3A–D). HepG2 spheroids always had somewhat irregular shape and surface with various recesses and ledges, as it is clearly seen on SEM image (Figure 3E). In contrast, HEK293 spheroids had a shape very close to spherical and relatively smooth surface (Figure 3F). We did not set out to study the growth features of HepG2 and HEK293 spheroids, since they are sufficiently described in literature, and we present here brief information on the growth and "appearance" of spheroids only to confirm the adequacy of our 3D models with published data.

#### 3.2.3. Electron Microscopic Features of HepG2 and HEK293 Spheroids

All published studies with HepG2 spheroids describe the “general appearance” of entire spheroids, noting the irregularity of shape and surface. Control of spheroids growth and analysis of their changes is carried out by means of light-optical observation of intravital characteristics as the perimeter length and square of spheroids, their roundness and compactness. Routine staining of paraffin sections mostly is used to show absence or presence of necrosis. Immunohistochemistry and fluorescence methods are increasingly introduced in recent years [11,12,13,14,15,45]. Published TEM data are scarce and usually present small portions of HepG2 cells [14,48]. Here we describe the ultrastructure of HepG2 spheroids with an emphasis on cell relationships and structure of spheroid outer surface and adjacent areas. We used a semithin section of each spheroid to choose a location for the pyramid, so we knew exactly which part of the spheroid was being explored in the TEM.

**Figure 4:**
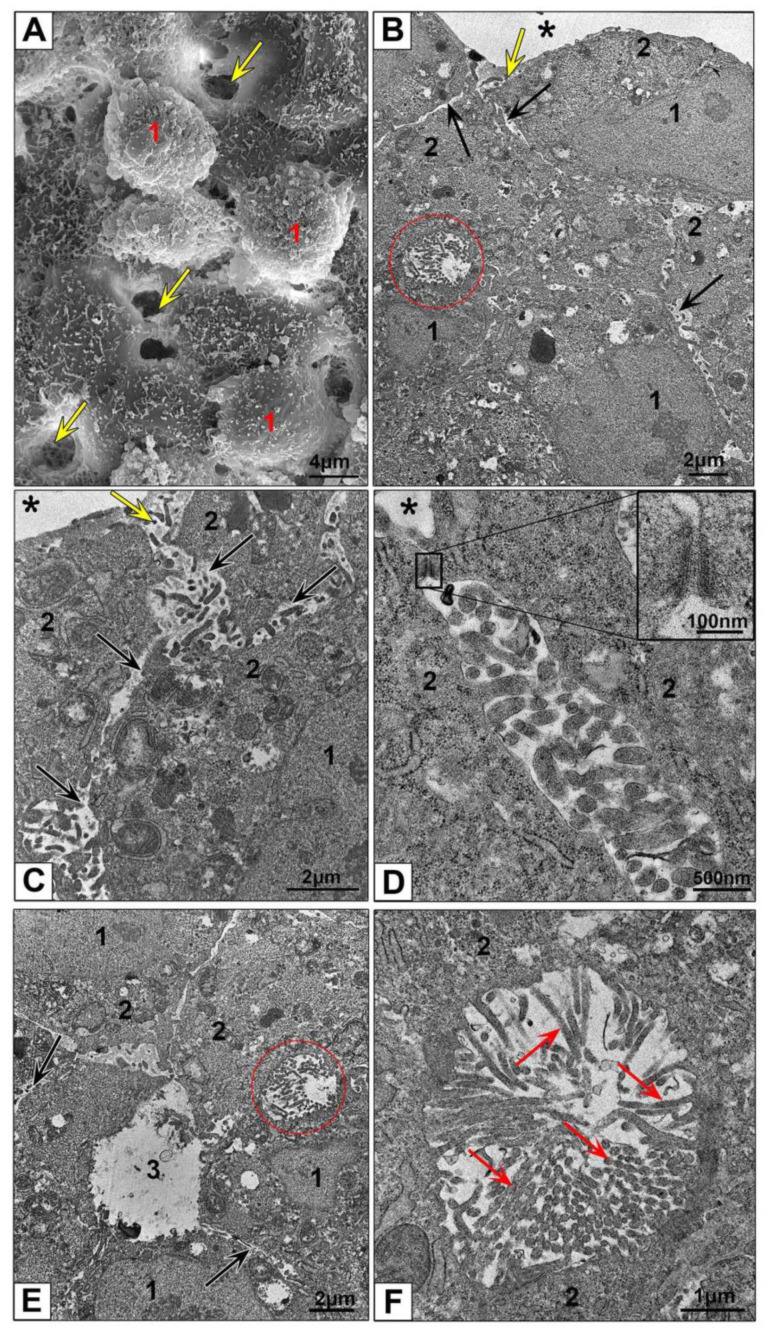
Ultrastructure of HepG2 spheroids. (**A**) Representative SEM image of spheroid surface. 1—bulging cells; arrows show openings on spheroid surface; (**B**–**D**) HepG2 spheroid periphery; (**E**) area at a distance of 50 µm from the surface; (**F**) bile capillary with microvilli. 1—nucleus; 2—cytoplasm; 3—lumen of cross-sectioned psedosinusoid; asterisks show external space; ovals show bile capillaries; yellow arrows show a space between hepatocytes (openings in SEM); black arrows show a space between lateral surfaces of neighboring cells; red arrows show microvilli; a frame shows desmosome between cells, and insert shows this desmosome at high magnification. (**B**–**F**) Ultrathin sections, TEM.

SEM examination of the HepG2 spheroids revealed an uneven surface resembling a “lunar landscape” with some bulging cells and "craters" extending into the interior of spheroid (Figure 4A). “Craters” looked as openings between spheroid cells and were randomly located on spheroid surface. Surface of the cells was covered with many small outgrowths.

Analyzing the results obtained by light microscopy, SEM and TEM we found that the openings are expansions of the space between the lateral surfaces of neighboring cells, serving as an “entrance” into the spheroid (Figure 4B,C and Figure S1). This “entrance” continued in the form of a space expanded up to one µm between the lateral surfaces of cells, covered with cytoplasmic outgrowths of different lengths and shapes. It should be noted that these outgrowths were distinctly different from microvilli on the surface of bile capillaries (Figure 4C–F). The openings were not formed by all cells: the “entrance” between the lateral cell surfaces could be closed with desmosomes (Figure 4D). Observed patterns suggest that cell basal membranes form outer surface of HepG2 spheroids contacting with culture medium and receiving the nutrients and external influences. Presence of the “pores” on surface of HepG2 spheroids were noted in SEM and TEM earlier [48].

Hepatocytes are epithelial cells with unique type of polarization, which sets a direction of their morphology and function, determines formation of bile capillaries by the apical surface, and retrieval of metabolites and other substances from sinusoid blood by basolateral surfaces [11,49]. In the liver, sinusoids formed by endothelial cells provide blood flow that is cleared by hepatocytes. Inside the spheroids we observed empty spaces between HepG2 cells resembling the sinusoids up to 2–2.5 µm width (Figure 4E and Figure S2). This similarity allowed us to designate these spaces as “pseudosinusoids”. It is unknown whether pseudosinusoids form a common network that permeates the spheroid; a special research is required to establish this.

Bile capillaries (bile canaliculi) formed by hepatocyte apical plasmalemma are bright morphological feature of hepatic tissue [11]. Bile capillaries are clearly visible in groups of HepG2 cells in monolayer (Figure 2A,B) and in ultrathin sections of HepG2 spheroids (Figure 4B,E,F and Figure S3). These structures are easily differentiated by the presence of microvilli on their surface, and could be found in various parts of spheroid, there are no visible ordering in their location. Analysis of the data obtained by TEM, SEM and light microscopy, clearly indicates that bile capillaries never come to the surface; they are "hidden" inside the spheroids. Correct identification of bile capillaries in HepG2 spheroids by TEM was reported in [14], and some published TEM studies of HEpG2 spheroids demonstrated pseudosinusoids instead bile capillaries [48,50].

Thus, the spheroids formed by HepG2 cells maintain typical for liver histology parameters: separation of the plasmalemma into apical and basolateral parts, formation of bile capillaries and pseudosinusoids. It is important that HepG2 spheroids face the environment with cell basal plasmalemma, which provides contact of hepatocytes with blood components in the liver. This feature of HepG2 spheroids should be taken into account when studying the effects of various preparations, including nanoparticles.

**Figure 5:**
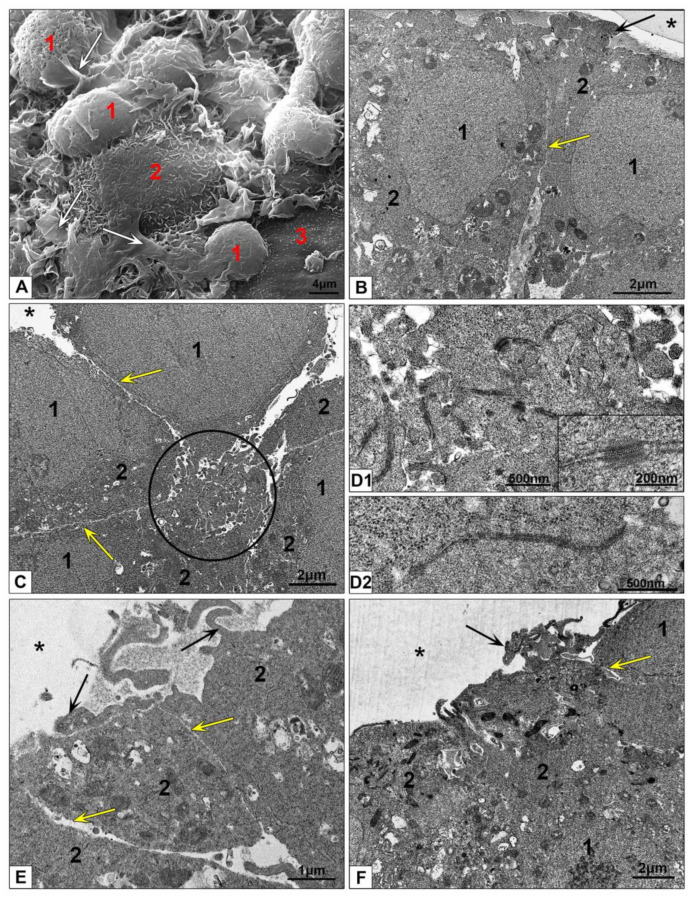
Ultrastructure of HEK293 spheroids. (**A**) Representative SEM image of spheroid surface. 1—cell body; 2—cell surface with small microvilli; 3—flat cell surface; white arrows show flat folds. (**B**–**F**) Cells at the periphery of spheroid, ultrathin sections. 1—nucleus; 2—cytoplasm; asterisks show external space; circle shows a conglomerate of apical outgrowths; arrows show outgrowths protruding in external space; yellow arrows show narrow space between lateral cell surfaces. (**D1**) Outgrowth conglomerate at higher magnification, the insert shows desmosomes; (**D2**) this photo presents a structure similar to apical tight junction.

Examination of HEK293 spheroids in a SEM revealed a significantly flatter surface than those in HepG2 spheroids due to absence of “craters” (Figure 3E,F, Figure 4A and Figure 5A). Thin flat folds of plasmalemma indicating macropinocytosis were visible between bulging cell bodies. Small cytoplasm protrusions were present on cell surface in different amount, some cells had smooth surface (Figure 5A). Different appearance of cell surface could reflect different functional state of the cells in HEK293 spheroids.

HEK293 cell were isolated from human embryo kidney and transformed with sheared DNA of adenovirus type 5 [42]. The obtained cell line was considered as epithelial cells. Presence of morphologically visible tight junctions in HEK293 monolayer were not reported [51,52], although expression of markers of epithelial tight junctions (zonula occludens) including ZO-1 and occludins was detected [53]. We did not observe tight junctions in monolayer HEK293 cells (Figure 2C,D).

Ultrathin sections of HEK293 spheroids showed groups of pyramidal cells (Figure 5C,E) connected to each other with narrow apical parts, which formed a conglomerate of interlaced cytoplasmic outgrowths bound by desmosomes and structures similar to tight junctions (Figure 5(D1,D2)). The conglomerates were observed throughout entire thickness of spheroids, not only at the periphery. On the ultrathin sections, these conglomerates looked like a disheveled skein of ribbons with ends sticking out in different directions. The center of conglomerate looked solid; there were no signs of a lumen formation, as in bile capillaries or glandular acini. We propose that apical cytoplasmic conglomerates organize HEK293 cells into groups, reflecting non-complete (without a lumen) formation of epithelial tube, determined by “columnar” polarization typical for of non-hepatic epithelia [39,41]. Thus, it is clear that cultivation of HEK293 cells in the 3D system induced formation of typical for epithelia structures (tight junctions and desmosomes) that were absent in the monolayer of these cells.

Surface of HEK293 spheroids was formed by basal plasmalemma of the cells, which was flat or covered with small protrusions. Some cells showed large outgrowths protruding in external space usually located near cell lateral borders (Figure 5B,E,F). It is obvious that formation of these structures is associated with the ability of HEK293 cells to macropinocytosis, which we noted on a monolayer culture. Similar structures were not observed in cells of HepG2 spheroid.

The lateral surfaces of spheroid cells were usually smooth and separated by narrow gaps led deep into the spheroid, widening of these gaps were observed in area of apical conglomerates (Figure 5B,C,E). Lateral plasmalemma of neighboring cells did not form interdigitations.

The results we obtained showed that cultivation of HepG2 and HEK293 cells in non-adhesive conditions in full media and without scaffold provide formation of spheroids; their morphological characteristics reflect structural polarization of epithelium in maternal organ. While HepG2 cells form bile capillaries and pseudosinusoids, HEK293 cells in these conditions are unable create complete structural units of epithelium with “columnar” polarization. Interestingly, the cells of both cultures were exposed to the culture medium by their basal plasmalemma, and the apical parts of the cells were inside spheroids.

### 3.3. Interaction of AuNPs, AuPEI-NPs and AuBSA-NPs with HepG2 and HEK293 Cells

#### 3.3.1. Ultrastructural Features of NPs Interaction with the Cells in Monolayer

Study of NPs uptake by HepG2 and HEK293 cells were performed in culture medium without serum during 4 h to prevent “corona” formation by serum proteins, because “corona” forms differently on differently charged NPs, and it can alter their behavior by unknown way [54,55].

**Figure 6:**
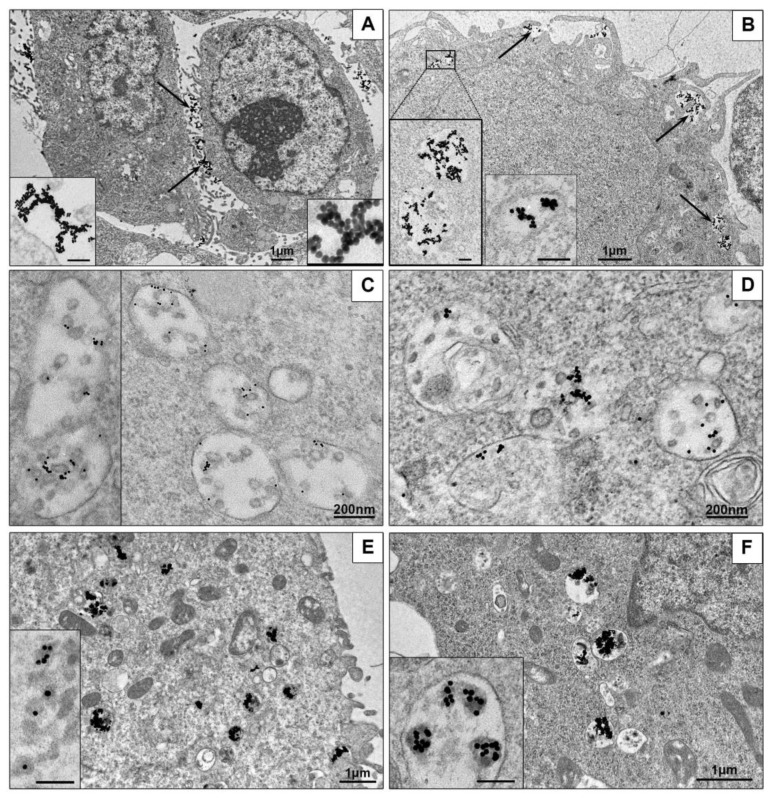
Representative TEM-images of NPs uptake by cells. (**A**) AuNPs on HepG2 cell surface are shown with arrows; inserts show AuNPs aggregations on plasmalemma at high magnification. 4 h incubation. (**B**) Penetration of AuNPs into HEK293 cells. Arrows show endocytosis-associated structures containing AuNPs; left insert shows two enlarged endosomes; right insert shows early endosome containing AuNPs. 30 min incubation. (**C**) AuBSA-NPs inside late endosomes of HepG2 cells. 4 h incubation. (**D**) AuBSA-NPs inside HEK293 cells. 4 h incubation. (**E**) AuPEI-NPs in endosomes of HepG2 cells; insert shows enlarged particles inside endosome, PEI looks as grey material around gold core. 1 h incubation. (**F**) AuPEI-NPs in endosomes of HEK293 cells; insert shows aggregates of AuPEI-NPs inside endosome. 1 h incubation. Bars in inserts correspond to 100 nm.

First, we examined interaction of AuNPs with HepG2 cells and unexpectedly found that all AuNPs remained on cell surface, no signs of the NPs penetration into cells were observed during 4 h of incubation (Figure 6A). In contrast, HEK293 cells readily internalized the same AuNPs, which were observed in structures associated with endocytosis after 15 min of incubation, and accumulated in cells up to 4 h (Figure 6B). At the same time, HepG2 cells actively engulfed AuNPs covered with PEI or BSA, as HEK293 cells did (Figure 6C–F). The accumulation of AuBSA-NPs was detected later than AuPEI-NPs (first AuBSA-NPs were found in both cell cultures after 2 h of incubation, the particles were dispersed in endosomes, and this reflects endocytosis of single particles (Figure 6C,D). The cells are able to accumulate many AuBSA-NPs in late endosomes (Figure S4), and it is easy to imagine the huge amount of NPs accumulated inside the cell, remembering that one ultra-thin section is about 70 nm thick, and the cell diameter is more than 10 microns. Previously long-term flotation of AuBSA-NPs was observed on HeLa cells [32], what is similar to current observation and obviously associated with particle negative net charge (Table 1). Penetration of AuPEI-NPs into both cell lines visually was highest, and numerous particles were found in endosomes after 15 minutes of incubation (Figure 6 E,F), obviously due to their positive net charge (Table 1). It is interesting, that all kinds of the NPs had different behavior inside endosomes of HepG2 and HEK293 cells: AuNPs formed loose aggregations of various shape, AuBSA-NPs localized individually; and AuNP-PEI formed compact aggregations or were located separately (Figure 6).

Undoubtedly, most interesting of our findings is absence of AuNPs uptake by HepG2 cells in contrast with active internalization of the same AuNPs covered with BSA or PEI. We did not find any study confirming or neglecting our results. Uptake of AuNPs (20 nm) by HepG2 cells was detected by inductively coupled plasma mass spectrometry [26], however, this method did not provide reliable results because it operates with a whole mass of the cells dissolved in 3% HNO3, so it is impossible say were AuNPs inside the cells or were they adsorbed on cell surface [25]. The TEM of ultrathin sections is the only method that unambiguously demonstrates the penetration of metal NPs into cells and allows identification of cell structures without a special labeling. Most published studies skip TEM examination of cell-NPs interaction. A number of studies provide images of the final stages of NPs accumulation in cells after several days of incubation; such data only confirm the presence of NPs in cells without bringing details of their penetration and interaction with cellular structures [22,23]. Meanwhile, understanding of the pathways of NPs internalization is necessary for biomedicine and nanotoxicology, current knowledge in the field is comprehensively analyzed in recent reviews, which noted advantages of TEM: possibility of direct and simultaneous visualization of NPs and cell structures on ultrathin sections [4,55,56].

**Figure 7:**
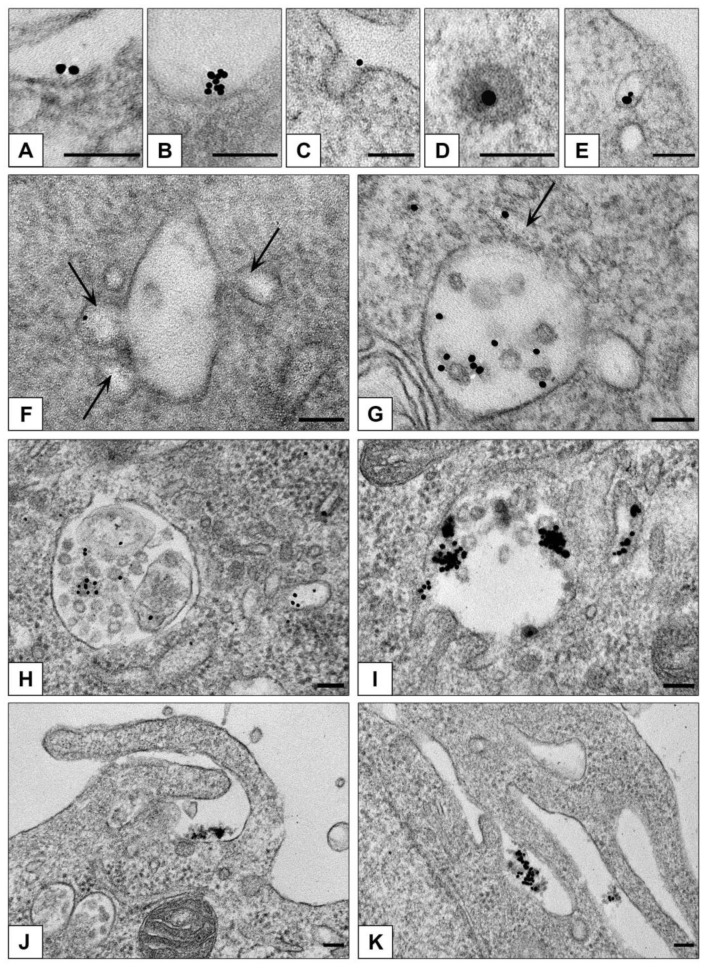
Representative images of NPs penetration into monolayer cells. Adsorption of single AuBSA-NP (**A**) and small cluster (**B**) of AuNPs on plasmalemma (HepG2); (**C**) coated pit containing AuNP and (**D**) coated vesicle containing AuPEI-NP (HEK293); (**E**) endocytotic vesicle containing AuBSA-NPs (HepG2). (**F**) Early endosome receives AuNP in vesicle; arrows show vesicles fusing with endosome body (HEK293). (**G**) AuNPs in endosome cavity, arrow shows NP in tubule (HEK293). (**H**) AuPEI-NPs in late endosome and a vesicle (HepG2). (**I**) AuNPs in late endosome (HEK293). (**J**,**K**) Macropinocytosis of AuPEI-NPs (HEK293). TEM, ultrathin sections. Bars correspond to 100 nm.

Our examination of ultrathin sections in TEM revealed that clathrin-mediated endocytosis is main way to enter both HepG2 and HEK293 cells for all studied types of NPs. Figure 7 demonstrates sequential steps of clathrin-mediated endocytosis: adsorption, transfer by vesicle to early endosome and accumulation in late endosomes. All types of NPs were observed only in membrane-bound structures (endosomes and lysosomes); no signs of NPs cytoplasmic localization were detected during 4 h of incubation. Our previous studies showed that AuNPs stay inside late endosomes and lysosomes of HeLa cells at least for 72 h [32].

HEK293 cells also showed the signs of macrpinocytosis of all studied NPs, cells developed long outgrowths and macropinocytic cups (Figure 7J,K). Morphological signs of macropinocytosis in HEK293 cells were identical to earlier published TEM data on this type of endocytosis, the authors noted independence of macropinocytosis and clathrin-mediated endocytosis [57,58,59].

Clathrin-mediated endocytosis was examined in HepG2 cells incubated with AuNPs coated with human ferrotransferrin (8 nm) or asialoorosomucoid (20 nm) in TEM and the data showed all subsequent steps of the endocytosis [60], identical to our images, however, author did not study interaction of HepG2 cells with AuNPs.

#### 3.3.2. Features of NPs Interaction with Spheroids Cells

**Figure 8:**
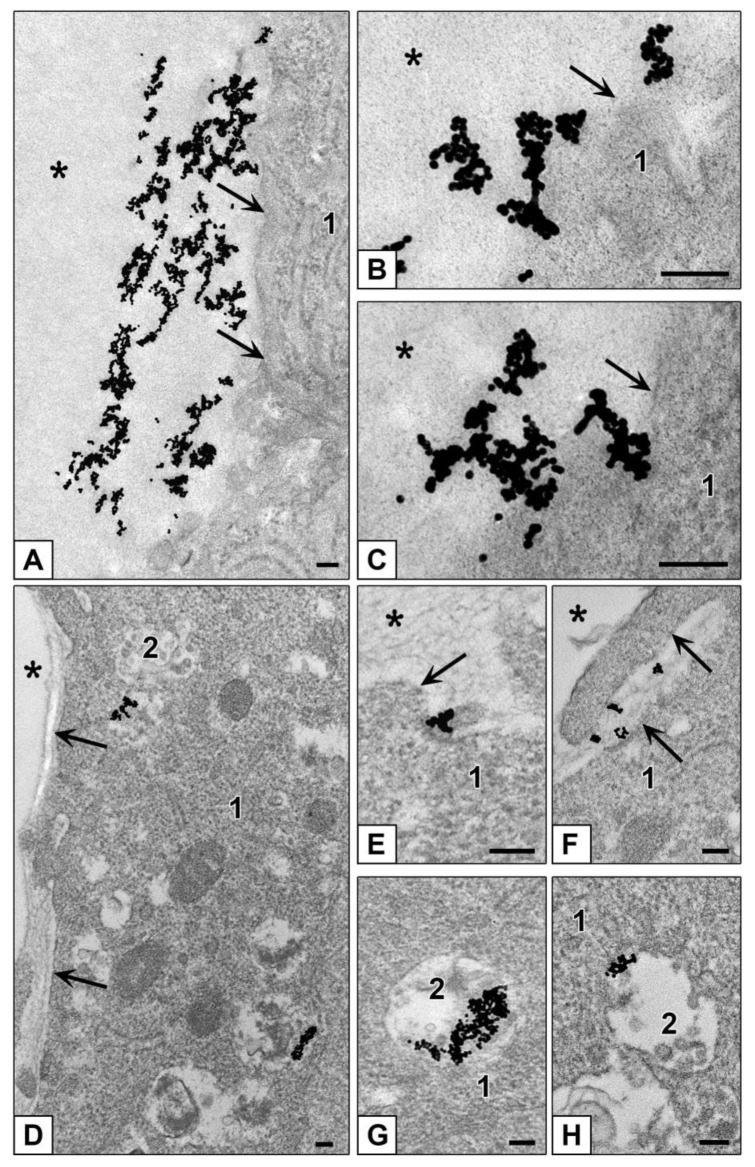
Interaction of AuNPs with cells of different spheroids. (**A**–**C**) adsorption of AuNPs on plasmalemma (HepG2 cells). (**D**–**H**) Penetration of AuNPs into HEK293 cells. (**E**) Adsorption of AuNPs on plasmalemma; (**F**) Macropinocytosis of AuNPs; (**G**,**H**) AuNPs in late endosomes. 1—cytoplasm; 2—late endosome; asterisks show external space and arrows show plasmalemma. Bars correspond to 100 nm.

Examination of ultrathin sections of HepG2 spheroids incubated with AuNPs showed inability of these cells to internalize these NPs, which were localized only on spheroid surface during 4 h (Figure 8A–C). At the same time, ultrathin sections of HEK293 spheroids showed signs of both clathrin-mediated endocytosis and macropinocytosis of AuNPs which were found mostly in late endosomes starting from 1 h of incubation (Figure 8D–H). HEK293 cells containing AuNPs were located in spheroid external zone (about 35–40 µm from the surface).

**Figure 9:**
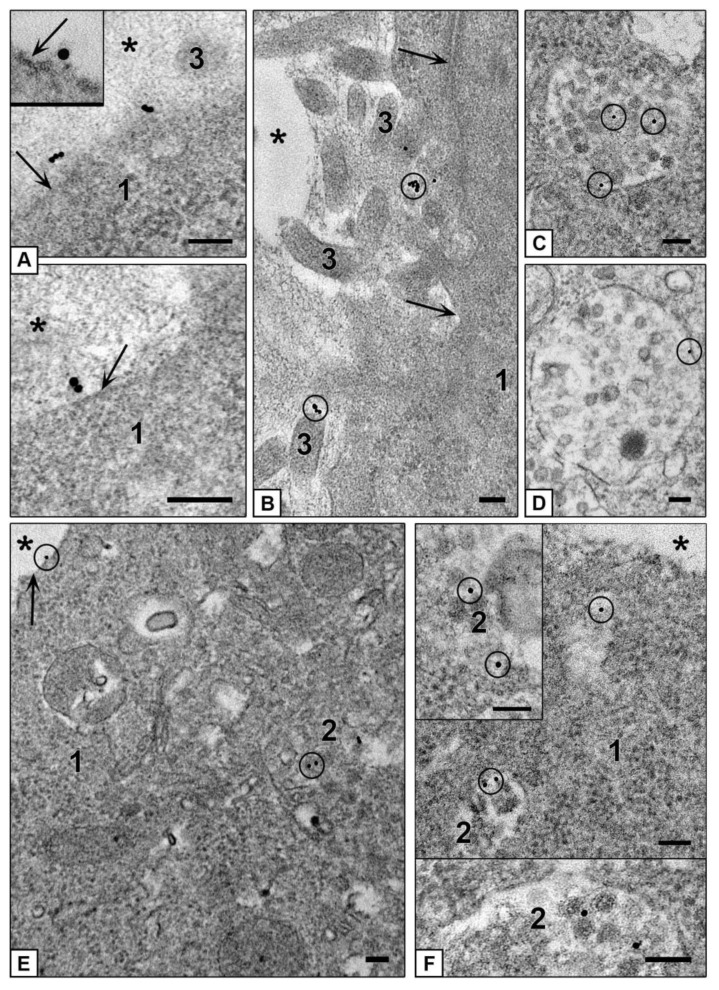
Interaction of AuBSA-NPs with cells of different spheroids. (**A**–**D**) Penetration of AuBSA-NPs into HepG2 cells. (**A**,**B**) adsorption of NPs on plasmalemma, insert shows NP adsorption at high magnification. (**C**,**D**) AuBSA-NPs in late endosomes. (**E**,**F**) Penetration of AuBSA-NPs into HEK293 cells. (**E**) NPs on plasmalemma and in late endosome. (**F**) AuBSA-NPs in late endosomes and in a vesicle. 1—cytoplasm; 2—late endosomes; 3—cell outgrowths; asterisks show external space and arrows show plasmalemma. Bars correspond to 100 nm.

Thus, both HepG2 and HEK293 cultures in form of spheroid (3D-culture) kept the character of interaction with AuNPs observed in their monolayers. The same phenomenon occurred during incubation with AuBSA-NPs: ultrathin sections of both HepG2 and HEK293 spheroids showed adsorption of NPs on plasmalemma and presence of individual particles in late endosomes (Figure 9). The cells containing AuBSA-NPs were located in external zone (about 35–40 µm from the surface) of HepG2 and HEK293 spheroids.

**Figure 10:**
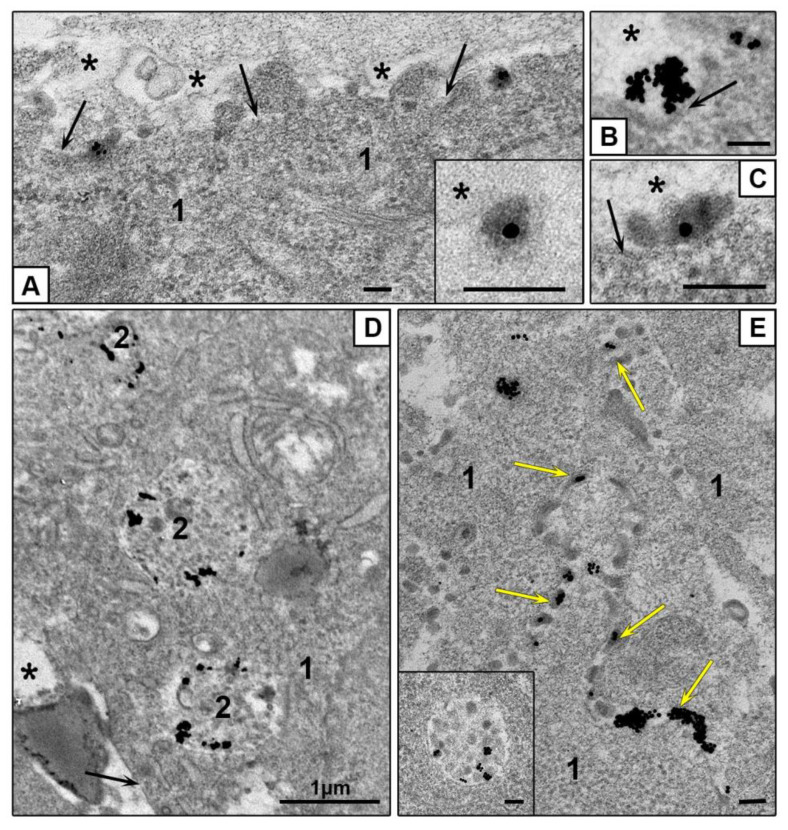
Interaction of AuPEI-NPs with cells of different spheroids. (**A**) Adsorption of AuPEI-NPs on HepG2 cell plasmalemma. Images in insert, (**B**,**C**) show NP adsorption at high magnification, HepG2 cells. (**D**) AuPEI-NPs in late endosomes of HepG2 cells. (**E**) Penetration of AuPEI-NPs into HEK293 cell, insert shows NPs in late endosome at high magnification, yellow arrows show NPs between spheroid cells. 1—cytoplasm; 2—late endosomes; asterisks show external space and arrows show plasmalemma. Bars correspond to 100 nm.

AuPEI-NPs were observed on basal plasmalemma of cells forming outer surface of HepG2 and HEK293 spheroids, and in the spaces between lateral cell surfaces, and in pseudosinusoids of HepG2 spheroids (Figure 10A–D). As in the case of a monolayer, AuPEI-NPs more actively penetrated the cells than other studied NPs and accumulated in late endosomes of both HepG2 and HEK293 cells (Figure 10D,E). AuPEI-NPs visually were more abundant in a tissue of HepG2 and HEK293 spheroids than AuBSA-NPs, however, their penetration depth was similar: 35–40 µm. Thus, positive net charge of AuPEI-NPs did not influence the depth of NPs penetration into spheroids, although it increased their accumulation inside spheroids, as well as in monolayer HepG2 and HEK293 cells.

## 4. Discussion

HepG2 cells cultured in 2D- and 3D-forms are used as experimental model in various studies related with hepatic functions and pathology, drug delivery and safety, toxicology and others [11,12,14,15]. Such a wide range of applications is due to the unique properties of this cell line, which reproduces structural and functional features of the liver, in particular, in 3D-form (spheroids), which is reviewed in [7,9,11]. Liver hepatocytes and HepG2 cells possess unique structural and functional polarity, it was interesting to compare their features with another cell line, representing “standard columnar” polarity, and we used HEK293 cell line for that. Using the TEM, we compared not only morphology of HepG2 and HEK293 cells in monolayers and spheroids, but also their interaction with three types of gold NPs differing by a coating nature, hydrodynamic size, and net charge.

In monolayer, HepG2 cells formed bile capillaries with typical microvilli and tight junctions, thereby showing liver-specific features. In contrast, HEK293 cells did not show any tissue-specific signs. In spheroids, both HepG2 and HEK293 cells formed outer surface by basal plasma membrane, which contacts with exterior environment (culture medium), and this finding can be important for understanding of the mechanisms of experimental influences. Our data show that spheroids consist of structural blocks, formation of which is specified by bile capillaries in case of HepG2 cells, and conglomerates of cell apical parts in case of HEK293. In HepG2 spheroids, blocks are separated by pseudosinusoids and intercellular spaces formed by lateral membranes, but more research is needed to find out whether pseudosinusoids form a common network within the spheroid. More research is also needed to understand how the blocks interact with each other; nevertheless, at present we can definitely say that the cells do not form shaped layers in HepG2 and HEK293 spheroids.

AuNPs are considered a promising platform for development of targeted nanomedicines and are extensively explored; however, there are many unknown details in their interaction with a cell [1,2,3,4,5,6]. We examined internalization of positively charged AuPEI-NPs, and negatively charged AuNPs and AuBSA-NPs with HepG2 and HEK293 cells in monolayers and spheroids to learn more about the mechanisms of their internalization. To our surprise, AuNPs did not penetrate HepG2 cells either in the monolayer or in the spheroids. Published TEM studies of AuNPs penetration into HepG2 cells were conducted in presence of serum [23], which contains proteins forming corona and so change a pattern of AuNPs interaction with a cell. However, our data corresponded to published TEM data that AuNPs (10 nm) did not penetrate hepatocytes in mouse liver, their uptake was detected in Kuppfer and endothelial cells (in contrast to AgNPs which penetrated the hepatocytes [4]. More research is needed to explain why hepatocytes and HepG2 cells "ignore" AuNPs, but an important point following from this observation is that cells can possess a selectivity for at least one type of the NPs. In connection with the selectivity of cells, it is pertinent to note the known effect of the size of AuNPs on penetration into cells. Thus, size-dependent accumulation of AuNPs (2–15 nm) coated with tiopronin was shown for MCF-7 breast cancer cells and their multicellular spheroids, and tumors in mice [61]. Different penetration rates were also reported for AuNPs (50 and 100 nm) coated with thiopronine in the same experimental MCF-7 cell models [62].

Interest to penetration of a drug into spheroid tissue is related with known block of the diffusion in tissue of solid tumors in vivo influencing drug effect [63]. Published studies show that the penetration of various NPs into HepG2 and other spheroids is limited to a depth of 20–50 µm, despite a fairly long incubation (for 24–72 h) [10,15,64]. The same values of penetration into HepG2 spheroids were reported for sorafenib [14]. Examination of MCF-7 spheroids treated with doxorubicin, revealed a dependence of penetration on spheroid sizes and ability of the drug to completely penetrate into the small-size spheroids [65]. Ability of doxorubicin for complete penetration into C3-HepG2 spheroid during 24 h was shown using confocal microscopy [45]. Our study showed that all studied NPs penetrated to a depth of 30–40 µm into HepG2 and HEK293 spheroids during 4 h of incubation. Limited penetration of NPs into spheroids is a “good” feature when a research is devoted to antitumor drugs, however, it may influence the results of toxicological and other studies.

Obviously, the use of spheroids in NPs research will expand and the methods of their cultivation and study of experimental effects will be increasingly improved. Presently, direct visualization of a NP in a cell and identification of cell structures is possible only by TEM of ultrathin sections. Currently, electron microscopy is overshadowed by other methods, faster and sometimes simpler, however, do not allowing to see directly nanoparticles in cells. Application of TEM allowed us to obtain a new data about features of HepG2 and HEK293 morphology in monolayer and spheroids, and clarify details of AuNPs, AuBSA-NPs and AuPEI-NPs uptake by these cells.

## 5. Conclusions

We compared ultrastructure of epithelial cells, which possess hepatocyte-type of polarization (HepG2) and columnar polarization (HEK 293) cultured in 2D- and 3D-forms (monolayer and spheroids). Monolayer HepG2 cells showed hepatic epithelia-specific morphological features, while HEK293 cells in monolayer did not show signs of epithelial tissue.

Cultivation of HepG2 and HEK293 cells on non-adhesive conditions led to formation of spheroids, a common feature of which was formation of spheroid outer surface by basal cell plasma membrane. This finding should be taken in account in experiments on drug delivery.

To examine interaction of different NPs with cells in monolayer and spheroids, we synthesized AuNPs (12.0 ± 0.1 nm in diameter, TEM data) and covered them with BSA and PEI. Values of hydrodynamic diameter were 17.4 ± 0.4; 35.9 ± 0.5 and 125.9 ± 2.8 nm for AuNPs, AuBSA-NPs and AuPEI-NPs, and Z-potential (net charge) values were −33.6 ± 2.0; −35.7 ± 1.8 and 39.9 ± 1.3 mV, respectively.

TEM study revealed inability of AuNPs uptake by HepG2 cells both in monolayer and spheroid form, while AuPEI-NPs and AuBSA-NPs were actively internalized via clathrin-mediated endocytosis, and this is an evidence for selectivity of HepG2 cells in respect to different NPs. At the same time, AuNPs actively penetrated the HEK293 cells in monolayer and spheroids.

Our data showed that the presence of a protein or polymer corona affects the behavior of NPs in endosomes after their endocytosis: AuBSA-NPs remained dispersed, while AuPEI-NPs were fused and formed aggregates. These features could influence drug release from an endosome, and so deserve attention when studying efficacy of drug delivery.

We did not observe appreciable distinctions in mechanisms of all studied NPs interaction with HepG2 and HEK293 cells in monolayer and spheroids. This observation is important for planning of different experiments because allow choosing cell monolayers or spheroids as experimental model, depending on a task. Thus, to know a depth of drug penetration, only spheroids are suitable, and for studies of drug-cell interaction mechanisms monolayers can be used. Undoubtedly, the use of multicellular spheroids as a 3D model of tumors and organs provides additional and “useful” opportunities for studying nanomedicine preparations in comparison with the cell monolayer.

## Figures and Tables

**Table 1:** Physicochemical characteristics of AuNPs, AuPEI-NPs and AuBSA-NPs.

Sample	Zeta-Potential (mV)	Polydipersity Index	Hydrodynamic Diameter (nm)
AuNPs	−33.6 ± 2.0	0.145 ± 0.006	17.4 ± 0.4
AuMUA-NPs	−53.8 ± 3.4	0.288 ± 0.001	46.0 ± 2.2
AuMUA-PEI-NPs	39.9 ± 1.3	0.258 ± 0.008	125.9 ± 2.8
AuBSA-NPs	−35.7 ± 1.8	0.212 ± 0.008	35.9 ± 0.5

## Supplementary Materials

The following are available online at https://www.mdpi.com/2079-4991/10/10/2040/s1, Figure S1: “Openings” on surface of HepG2 spheroids in SEM and semithin sections, Figure S2: Pseudosinusoids in ultrathin sections of HepG2 spheroids, Figure S3: Periphery of HepG2 spheroid, Figure S4: Accumulation of AuBSA-NPs in HepG2 cells.
