# Supplementary material for: Ultrastructural Features of Gold Nanoparticles Interaction with HepG2 and HEK293 Cells in Monolayer and Spheroids
Source: Nanomaterials (Basel). 2020 Oct 16;10(10):2040. doi: 10.3390/nano10102040 (PMC7650816; doi:10.3390/nano10102040)
Supplement: Supplementary file 1 [file nanomaterials-10-02040-s001.zip › nanomaterials-928800-supplementary.doc]

**Supplementary Materials for article**

Ultrastructural Features of Gold Nanoparticles Interaction with HepG2 and HEK293 Cells in Monolayer and Spheroids

Boris Chelobanov ^†^, Julia Poletaeva ^†^, Anna Epanchintseva, Anastasiya Tupitsyna, Inna Pyshnaya and Elena Ryabchikova *

Institute of Chemical Biology and Fundamental Medicine, Siberian Branch of Russian Academy of Science, Lavrent’ev av., 8, 630090 Novosibirsk, Russia; boris.p.chelobanov@gmail.com (B.C.); fabaceae@yandex.ru (J.P.); annaepanch@gmail.com (A.E.); aysa@ngs.ru (A.T.); pyshnaya@niboch.nsc.ru (I.P.)

*****	Correspondence: lenryab@yandex.ru; Tel.: +7-383-363-51-63

†	These authors contributed equally to this work.

**Figure S1**. “Openings” on surface of HepG2 spheroids in SEM (upper row) and in semithin sections stained with Azur 2 (bottom row). The arrows show the space between the hepatocytes, which penetrates deep into the spheroids. Asterisks show external space.

**Figure S2**. Pseudosinusoinds (1) on ultrathin sections of HepG2 spheroids. 2 – nucleus; 3 – cytoplasm. Arrows show a space between cell lateral surfaces. TEM.

**Figure S3**. Periphery of HepG2 spheroid. 1 – nucleus; 2 – cytoplasm; 3 - bile capillary with microvilli; asterisk shows external space.

**Figure S4**. Accumulation of AuBSA-NPs in HepG2 cells during 4 h of incubation. Photo demonstrates a portion of cytoplasm containing many late endosomes (multivesicular bodies) at different stages of their “maturation” to lysosomes (1 and 2). 3 – structures of Golgi complex; red circles show some endosomes, containing AuBSA-NPs.
